# Cocaine‐Specific Effects on Exosome Biogenesis in Microglial Cells

**DOI:** 10.1007/s11064-021-03231-2

**Published:** 2021-02-08

**Authors:** Sanjay Kumar, Brennetta J. Crenshaw, Sparkle D. Williams, Courtnee’ R. Bell, Qiana L. Matthews, Brian Sims

**Affiliations:** 1 Department of Pediatrics/Division of Neonatology and Center of Glial Biology in Medicine at the University of Alabama School of Medicine, UAB Women and Infant Center, University of Alabama, 1700 6th Ave South, Birmingham, AL 35294 USA; 2 Microbiology Program, Department of Biological Sciences, College of Science, Technology, Engineering and Mathematics, Alabama State University, Montgomery, AL 36104 USA

**Keywords:** Cocaine, Exosomes, BV2 microglia, Heat shock proteins, Lipids, rab GTPases

## Abstract

Cocaine is a highly addictive stimulant and a well-known drug, with multiple effects on physiology. Cocaine can have direct effects on all cell types in the brain, including microglia. Microglia can be activated by other conditions, such as infection, inflammation, or injury. However, how cocaine regulates microglia and the influence of cocaine on microglial-derived exosomes remains unknown. Exosomes are nanovesicles that are responsible for intercellular communications, signaling, and trafficking necessary cargo for cell homeostasis. In this study, we hypothesized that cocaine affects exosome biogenesis and composition in BV2 microglial cells. BV2 microglial cells were cultured in exosome-depleted RPMI-1640 media and were treated according to the experimental designs. We observed that cell viability decreased by 11% at 100 µM cocaine treatment but was unaffected at other concentrations. After treatments, the exosomes were isolated from the condition media. Purified exosomes were characterized and quantified using transmission electron microscope (TEM) and nanoparticle tracking analysis (NTA). By NTA, there was a significant decrease in particles/mL after cocaine treatment. There was a 39.5%, 58.1%, 32.3% and 28.1% decrease in particles/mL at 100 nM, 1 μM, 10 μM and 100 μM cocaine, respectively. The characterization of exosomes and exosomal protein was performed by western/dot blot analyses. Tetraspanins CD11b, CD18 and CD63 were relatively unchanged after cocaine treatment. The heat shock proteins (Hsps), Hsp70 and Hsp90, were both significantly increased at 10 μM and 100 μM, but only hsp70 was significantly increased at 10 nM. The Rab proteins were assessed to investigate their role in cocaine-mediated exosomal decrease. Rab11 was significantly decreased at 10 nM, 100 nM, 1 μM, 10 μM and 100 μM by 15%, 28%, 25%, 38% and 22%, respectively. Rab27 was decreased at all concentrations but only significantly decreased at 100 nM, 1 μM and 100 μM cocaine by 21%, 24% and 23%, respectively. Rab35 had no significant changes noted when compared to control. Rab7 increased at all cocaine concentrations but only a significant increase in expression at 100 nM and 10 μM by 1.32-fold and 1.4-fold increase. Cocaine was found to alter exosome biogenesis and composition in BV2 microglial cells. Western and dot blot analyses verified the identities of purified exosomes, and the specific protein compositions of exosomes were found to change in the presence of cocaine. Furthermore, cocaine exposure modulated the expression of exosomal proteins, such as Hsps and Rab GTPases, suggesting the protein composition and formation of microglial-derived exosomes were regulated by cocaine.

**Supplementary Information:**

The online version of this article (doi:10.1007/s11064-021-03231-2) contains supplementary material, which is available to authorized users.

## Introduction

Microglia are considered to be the most potent immune cells in the central nervous system (CNS) [1]. Microglia are activated during stress, inflammation, infections, and conditions that result in cellular damage, leading to the phagocytosis of damaged cells and the secretion of various cytokines [1–3]. Microglia survey and monitor the brain for harmful substances and pathogenic agents such as bacteria [1–3]. Upon activation, these cells transform from a ‘resting state’ to an ‘active state’ [3], during which microglia undergo changes in mobility and cellular morphology. Glial cell activation leads to the expression of specific receptors on their surfaces [4, 5]. In the ‘active state’, microglial cells release cytokines and demonstrate enhanced phagocytic functions [4, 5]. Microglia, like other cells, utilize exosomes for intercellular communications [6–8]. It is unclear if exosome biogenesis changes when microglia are activated.

Exosomes are unique structures that contain multiple proteins, lipids, microRNA, and RNA molecules [6–15]. They are usually 30–150 nm in diameter and express specific proteins such as alix, tetraspanins, integrins, Tsg101, heat shock proteins (Hsps), and Rab GTPases [6–15]. Exosomal membranes are enriched in specific lipid compounds, such as sphingomyelin, phosphatidylserine, phosphocholine, and cholesterol [12–20]. Exosomes have also demonstrated the ability to regulate cell waste by acting as cargo vessels [21]. Exosomes originate from multivesicular bodies (MVBs), through the inward budding of endosomal membranes, and are released when MVBs fuse with the plasma membrane. All body fluids (such as breastmilk, urine, blood, and plasma) have been shown to contain exosomes; however, their specific roles in different parts of the body remain unclear.

The regulation of exosome secretion by microglial cells is not well-understood; however, some studies have suggested that cytokines are involved in the regulation of exosome secretion and formation. Drugs of abuse, such as cocaine, have been shown to cross the blood–brain barrier and be a potent activator of microglia [22]. Several studies have demonstrated that F-actin is disrupted in endothelial cells and a concomitant decrease in expression of tight junction proteins leading to weakening of the blood–brain barrier [23–26]. Cocaine is one of the most used illicit drugs in the United States. Cocaine abuse results in a variety of CNS disorders, including an increased risk of stroke, seizures, cognitive impairment, depression, and, in extreme cases, death [27, 28]. Studies have demonstrated that cocaine administration can enhance the expression of cytokines/chemokines and adhesion molecules, through the binding of cocaine with its cognate receptor, which are expressed on a variety of cells [22, 29, 30], and these changes could result in altered exosomal production.

Studies have demonstrated cocaine-specific effects on microglial activation such as the release of brain-derived neurotrophic factor, other growth factors, and associated regulation of microRNA [22, 29, 30]; however, the effects of cocaine on exosome biogenesis and composition have not been studied. Therefore, in the present investigation, we aimed to test the effects of cocaine on the biogenesis and composition of BV2 microglial-derived exosomes. This investigation is the first of its sort and could help improve our comprehension of exosomal biology.

## Materials and Methods

### Cell Culture and Cocaine Exposure

Microglial (BV2) cells were grown in complete medium (Roswell Memorial Park Institute-1640 (RPMI-1640) medium (Fisher Scientific, Hampton, NH, USA), supplemented with 10% fetal bovine serum (FBS), containing 1X L-glutamine, 1% penicillin/streptomycin, and 0.05% Amphotericin-B (Fisher Scientific, Hampton, NH, USA), at 37 °C, in a 5% CO_2_ atmosphere. These cells were a generous gift from Dr. Harald Neumann at the University of Bonn LIFE and Brain Center in Bonn, Germany [31]. BV2 microglial cells were plated at a density of 2 × 10^6^ cells/dish and allowed to acclimatized overnight before cocaine (Sigma, St. Louis, MO, USA) treatments. The medium from each dish was removed and replaced with either exosome-free RPMI-1640 media only (control treatment) or exosome-free RPMI-1640 media containing 10 nM, 100 nM, 1 μM, 10 μM, or 100 μM cocaine for 24 h. All experiments were performed using 3–5 independent experiments.

### Trypan Blue Exclusion

To test cell viability, the trypan blue exclusion method was utilized. BV2 cells were harvested and centrifuged at 500 revolutions per min (rpm), for 5 min, at 4 ºC. The supernatant was discarded, the cell pellet was resuspended in 1 mL complete medium, and 10 µL resuspended pellet was mixed with 10 µL trypan blue dye (Fisher Scientific, Hampton, NH, USA). After gentle mixing, 10 µL of the cells mixed with trypan blue were loaded into a hemocytometer to perform a live/dead cell count. The resulting values were plotted on a graph to examine differences in the numbers of live and dead cells among the treatment groups. Viable cells were calculated using the following formula:

$${\text{Viable cells }} = \, \left[ {1.00 \, {-} \, \left( {{\text{Number}}\;{\text{of}}\;{\text{blue}}\;{\text{cells }} \div {\text{ Number}}\;{\text{of}}\;{\text{total}}\;{\text{cells}}} \right)} \right] \, \times \, 100.$$

### Microscopic Examination

To assess the cell morphology, microglial cells were exposed to 10 nM, 100 nM, 1 μM, 10 μM, and 100 μM cocaine for 24 h. After 24 h, the morphologies of the microglial cells were examined at ×10 magnification using an Invitrogen EVOS ™ FL system ™ (ThermoFisher Scientific, Waltham, MA, USA).

### Ultracentrifugation

To isolate and purify exosomes from cocaine-treated microglia, the media was carefully collected and centrifuged at 1300 rpm at 4 °C for 10 min, using a Sorvall 6000 refrigerated centrifuge (Sorvall. Ontario, Canada). The pellet was discarded, and the supernatant was centrifuged again at 39,000 rpm, at 4 °C for 10 min, and the resulting supernatant was filtered through a 0.22 μM filter and collected in ultracentrifuge tubes. The samples were then centrifuged at 10,800 rpm at 4 °C for 45 min, in an SW41T1 swinging bucket rotor, using a Beckmann Coulter Optima L-70 K Ultracentrifuge Beckman Counter, IN, USA. The exosome fraction was collected by the ultracentrifugation of the resulting supernatant at 32,000 rpm, in an SW41T1 swinging bucket rotor, for 70 min at 4 °C. The total protein levels in the exosome fraction were quantified using the Lowry protein quantification method.

### Transmission Electron Microscopy (TEM)

Exosomes were produced by incubating BV2 cells in exosome depleted-medium containing 10 nM, 100 nM, 1 μM, 10 μM, or 100 μM of cocaine and without cocaine (control) for 24 h. Freshly isolated BV2-derived exosomes were resuspended in PBS and diluted in 1:1 with 5% glutaraldehyde. Before loading sample on the EM-grids, carbon film coated mesh copper EM-grid were glow discharge at 50 mA for 20 s; thereafter, 7 µL exosomes suspension solution was loaded on the grid and incubated for 1 min at RT. Wick excess with a torn edge of a Whatman filter paper by wicking from below the grid was done in order to pull the sample towards the grid rather than away from it. Samples were immediately stained with 7 µL of filtered Uranyl acetate (UA) solution on the surface of the EM-grid. After 15 s excess UA solution was removed and samples were observed under transmission electron microscope (TEM) Tecnai 120 kV (FEI, Hillsboro, OR) at 80 kV within 24 h as compared to the negatively stained grids. Digital images were captured with a BioSprint 29 CCD Camera (AMT, Woburn, MA).

### Nanoparticle Tracking Analysis

To assess the sizes and numbers of exosome particles per mL solution, nanoparticle tracking analysis (NTA) was performed, using a NanoSight-LM10 (Malvern Instrument, Inc., Malvern, UK). The samples were diluted in 1 × phosphate buffer saline (PBS) and loaded into a 0.3 mL disposable syringe. The NTA analyzes samples based on the principle of Brownian particle movement. The mean values for five independent experiments were recorded and processed for each reading frame.

### Western and Dot Blot Analysis

To examine the expression of tetraspanin, adhesion molecules, Hsps, and Rab GTPases, western and dot blot analyses were performed, using 60 µg/well for western blot and 5 µg total protein per dot, after boiling at 99 °C for 5 min. Proteins were transferred onto PVDF membranes at 15 V for 1 h and for dot blot membranes were allowed to dry for 5–10 min, then blocked with Pierce Fast Blocker, containing 0.09% Tween-20 for 5–15 min at room temperature (RT) on a shaker. Then, membranes were hybridized with the following primary antibodies against tetraspanin and membrane molecules, for 1 h, at RT: Cluster of differentiation (CD)11b (0.1 µg/mL), CD18 (1:500), CD63 (0.5 µg/mL), Calnexin (1:5000), Hsp70 (1:1,000), Hsp90 (1:1000), Rab7 (1 µg/mL), Rab11, (0.5 µg/mL), Rab27A (0.5 µg/mL), and Rab35 (1:750) (all from Fisher Scientific, Hampton, NH, USA). Membranes were washed with 1 × Tris-buffer-saline (TBS) buffer, containing 0.09% Tween-20 (TBST-20), for 3x-10 min each wash. The appropriate horseradish peroxidase-conjugated secondary antibodies (Fisher Scientific, Hampton, NH, USA), goat anti-rabbit (1:1000), goat anti-mouse (11,000), or goat anti-hamster (1:5000), were incubated with the membranes in 1–2% non-fat milk solution in TBST-20 buffer for 1 h at RT. Membranes were washed three times with TBST-20, for 10 min per wash, and developed using an Invitrogen Novex ECL chemiluminescence liquid substrate kit (ThermoFisher Scientific, Waltham, MA, USA). The signals were detected on X-ray and a Bio-Rad ChemiDoc XRS^+^ system (BioRad, Hercules, CA, USA).

### Lipid Assay

Total lipid, total cholesterol, phospholipid, sphingolipid, phosphatidylcholine (Cell BioLabs, Inc., San Diego, CA, USA), and phosphatidylserine (BioVision, Milpitas, CA, USA) and levels were determined in the exosome fractions using a fluorometric assay. For each assay, 30 µg isolated exosome fraction from control or cocaine-treated samples was added to each well, in duplicate, using n = 3–5 of standard, lipid cholesterol, phospholipid, sphingolipid, phosphatidylserine, and phosphatidylcholine. To each well, 100 µL of the reaction reagent was added, and the well contents were mixed thoroughly. The plates were covered, protected from light, and incubated for 45–60 min at 37 °C, then read with a fluorescence microplate reader equipped for excitations in the 530–570 nm range and for emissions in the 590–600 nm range.

$${\text{Total}}\; {\text{lipid}}\; {\text{component}}=\left[\frac{{\text{sample}} \;{\text{corrected}}\; {\text{fluorescence}}}{\text{slope}}\right] {\text{sample}} \;{\text{dilution}}$$

### Statistical Analysis

Statistical analyses were performed using one-way analysis of variance (ANOVA) with Tukey post hoc analysis. Statistical significance is indicated by the mean ± SD as follows: p < 0.05 (*); p < 0.01 (**); p < 0.001 (***); and p < 0.0001(****).

## Results

### Cocaine Exposure Reduced BV2 Cell Viability

**Fig. 1:**
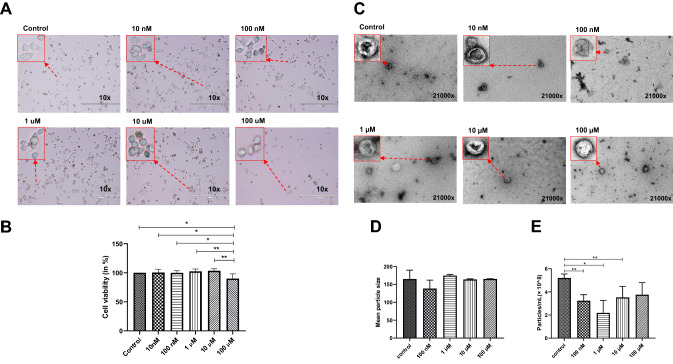
Cocaine-specific effects on BV2 microglial cell viability and the mean size and number of particles. BV2 microglial cells were treated with 10 nM, 100 nM, 1 µM, 10 µM, and 100 µM cocaine. Cells were grown in exosome-free medium and the cocaine was added for a maximum of 24 h. **a** Microscopy, **b** cell viability, **c** TEM, **d** mean particle size and **e** particle/mL. Mean size is shown in nanometers, and particle numbers are shown as 10^8^ per mL. Statistical significance is taken from 3 to 5 independent experiment in triplicates and indicated the mean of SD as follows: *p < 0.05; **p < 0.001; and ***p < 0.0001

To test the direct effects of cocaine on cellular viability, cells were treated with cocaine (10 nM, 100 nM, 1 µM, 10 µM, and 100 µM) and then assessed for cell morphology, under an inverted light microscope, and cell viability, using a trypan blue exclusion method, as previously described [32–34]. Our findings demonstrated that control cells (Fig. 1a) showed robust growth, as indicated by the cell culture surface at 24 h, whereas exposure to 100 µM cocaine caused morphological alterations, resulting in a more complete rounding of the cells (Fig. 1a). To further validate these findings, BV2 cell viability was assessed using the trypan blue exclusion method 24 h after cocaine was added, which was the duration of the experiment. Our findings suggested that cells treated with 100 µM cocaine showed reduced cell viability by 11% when compared with control cells and other experimental groups, such as the 10 nM, 100 nM, 1 µM, and 10 µM cocaine treatment groups (* p < 0.05 and ** p < 0.01) (Fig. 1b). These findings suggested that BV2 cell viability was affected at the highest cocaine concentration examined in this study (Fig. 1a and b; all individual data points can be observed in supplemental Figs. 1–5).

### Effects of Cocaine on Exosome Characteristics

Previous studies have demonstrated that almost all cell types, including bacteria, produce nanosized vesicles known as exosomes [35, 36]. Exosomes play important roles in cell-to-cell communication and signaling and can act as cargo vesicles, and altering these functions could have critical implications for cells. Therefore, we tested the effects of cocaine treatments on the mean size and particle numbers of exosomes per mL. BV2 cell-derived exosomes were purified, using ultracentrifugation, and the purified exosomes were evaluated for size using TEM and NTA (Fig. 1c and d), and the number of exosome particles per mL was also determined by NTA (Fig. 1e). Our findings suggested that the mean size was not affected by cocaine administration. Control mean size (164.83 ± 24.93 nm) was compared to 100 nM (138.37 ± 40.70 nm), 1 μM (174.64 ± 7.19 nm), 10 μM (163.74 ± 4.15 nm) and 100 μM (165.04 ± 1.77 nm) and no significant changes noted. However, the particle numbers per mL were significantly reduced after treatment comparing control (5.20 ± 0.35*10^8^ particles/mL) by 39.5% at 100 nM (3.23 ± 0.53*10^8^ particles/mL), by 58.1% at 1 µM (2.18 ± 1.08*10^8^ particles/mL), by 32.3% at 10 µM (3.52 ± 0.95*10^8^ particles/mL) and by 28.1% at 100 μM (3.75 ± 1.05*10^8^ particles/mL) of cocaine (Fig. 1e). Treatment with 100 µM cocaine did not result in a significant difference compared with the control (Fig. 1e).

### Cocaine-Specific Effects on Cell Membrane Proteins in Exosomes

**Fig. 2:**
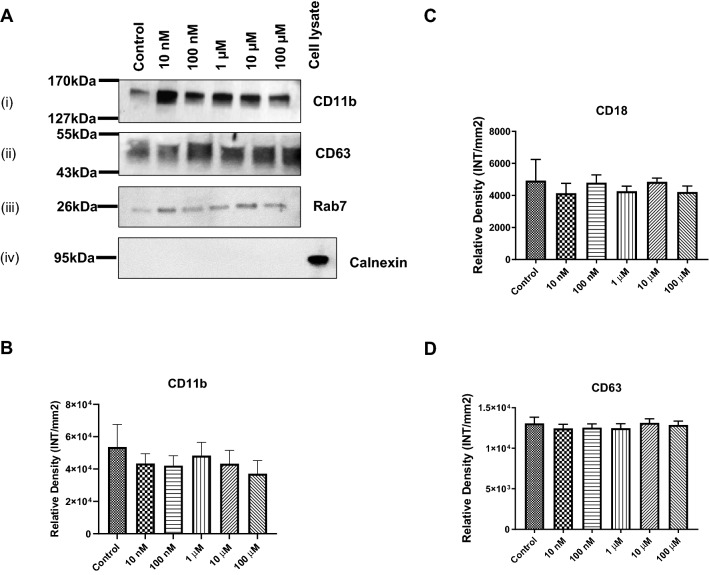
Cocaine-specific effects on cell membrane molecules. BV2 microglial cells were treated with cocaine (10 nM, 100 nM, 1 µM, 10 µM, and 100 µM) for 24 h, and the expression of cell membrane molecules in exosomes was determined using western and dot blot analysis. **a** representative western blots; (**i**) CD11b, (**ii**) CD63, (**iii**) Rab7 and (**iv**) Calnexin, **b** CD11b expression, **c** CD18 expression and **d** CD63 expression. Statistical significance is taken from 5 independent experiment in triplicates and indicated the mean of SD as follows: *p < 0.05; and **p < 0.001

The cell membrane is composed of several types of proteins, which act as a barrier and a communication platform, connecting the outside world to the intracellular control centers. Clusters of differentiation proteins, such as CD63 and CD81, are tetraspanin molecules that interact with a variety of cell surface proteins and intracellular molecules, inducing processes that include adhesion, motility, membrane organization, and signal transduction [37–41]. The transmembrane proteins CD11b (a surface marker for microglia, monocytes, and macrophages) and CD18 play critical roles in cellular adhesion [42]. Calnexin protein should only be in the cell but not in the exosomes. To examine the expression of these proteins following cocaine treatment, western/dot blot analyses of exosomal protein was performed. As it was shown in a representative Fig. 2a(i) expression of CD11b, 2A (ii) expression of CD63, 2A (iii) expression of Rab7, and 2A(iv) expression of Calnexin. We found that CD11b (Fig. 2b), CD18 (Fig. 2c), and CD63 (Fig. 2d) showed slightly decreased expression in exosomes after cocaine treatment; however, these changes were not significant when compared with the control. Furthermore, BV2 cell-derived exosomes showed low expression of CD81 (data not shown).

### Cocaine-Specific Effects on Hsp Expression in Exosomes

**Fig. 3:**
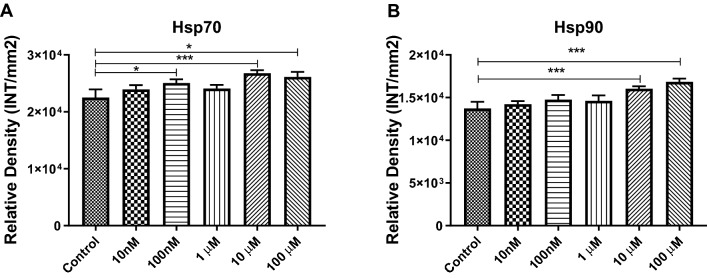
Cocaine-specific effects on Hsps in BV2 cells and exosomes. BV2 microglial cells were treated with cocaine (10 nM, 100 nM, 1 µM, 10 µM, and 100 µM) for 24 h, and the expression levels of was evaluated in in exosomes. **a** Hsp70 and **b** Hsp90 densities derived from dot blot. Statistical significance derived from 5 independent experiment in triplicates is indicated the mean of SD as follows: *p < 0.05; **p < 0.001; and ***p < 0.0001

Hsps, or stress proteins, are members of a highly conserved group of proteins found in all eukaryotes and prokaryotes, including bacteria. They act as molecular chaperones, assisting the proper folding/refolding of newly synthesized proteins [43, 44]. In addition, they play cytoprotective roles under stress and trauma conditions, and their expression levels increase many-fold when cells are exposed to drugs, heavy metals, and heat [43, 44]. HSPs can be found in exosomes; therefore, in the current investigation, the expression of Hsp70 and Hsp90 were evaluated using dot blot analysis. Our results indicated that exosomal Hsp70 expression were significantly increased after treatment with 100 nM (1.11 fold increase, p ≤ 0.05), 10 µM (1.19 fold increase, p ≤ 0.0001), and 100 µM (1.16 fold increase, p ≤ 0.05) cocaine compared with control (Fig. 3A), whereas Hsp90 (Fig. 3B) showed statistically significant increases in its expression after treatment with 10 µM (1.17 fold increase, p ≤ 0.0001) and 100 µM (1.22 fold increase, p ≤ 0.0001) cocaine when compared with the control (Fig. 3A and B), suggesting that Hsps are regulated by cocaine administration.

### Cocaine Modulates Rab GTPases in Exosomes

**Fig. 4:**
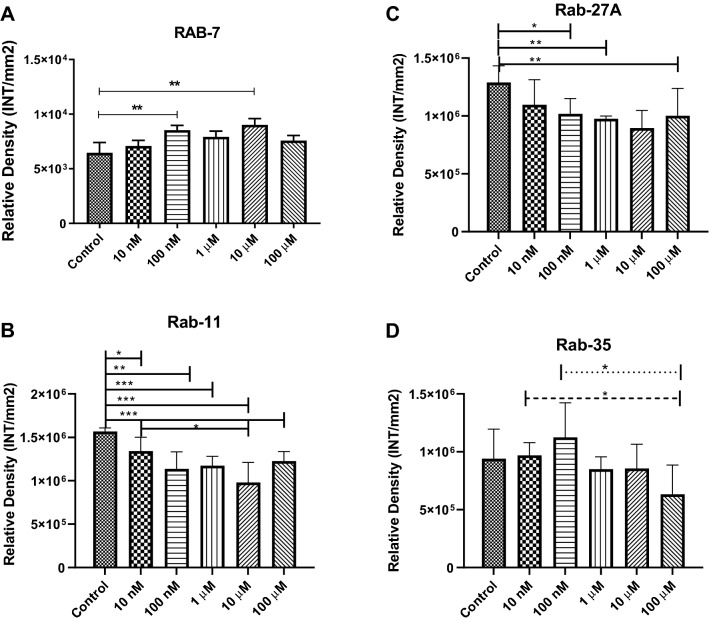
Cocaine-specific effects on Rab GTPases. To examine the expression of Rab GTPases in microglial cells, cells were incubated with 10 nM, 100 nM, 1 µM, 10 µM, and 100 µM cocaine for 24 h, and Rab protein expression levels were evaluated in BV2 cells and exosomes using dot blot analysis. **a** Rab7, **b** Rab11, **c** Rab27A and **d** Rab35 expression in BV2 cell-derived exosomes. Statistical significance derived from 5 independent experiment in triplicates is indicated the mean of SD as follows: *p < 0.05; **p < 0.001; and ***p < 0.0001

In this study, we showed that cocaine exposure altered the number of exosome particles per mL. To test whether this reduction in exosomal numbers was associated with Rab GTPases, we examined the expression levels of Rab7, Rab11, Rab27A, and Rab35 in BV2 cells and exosomes. Rab proteins belong to the Ras superfamily of small Rab GTPases [45]. Rab5 and Rab7 are present in the plasma membrane and early endosomes, and regulate vesicular trafficking during early endocytosis, whereas Rab11, Rab27A, and Rab35 are associated with protein sorting, secretion, and targeting [45–47]. Therefore, Rab proteins represent significant components of exosome biogenesis, sorting, and secretion machinery. Our experimental findings showed that Rab7 expression in exosomes increased after exposure to 100 nM (1.32 fold increase, p ≤ 0.001), and 10 µM (1.40 fold increase, p ≤ 0.001) cocaine compared with those in the control (Fig. 4a). Furthermore, Rab11 expression was downregulated significantly in exosomes after exposure to 10 nM (15% decrease, p ≤ 0.05), 100 nM (28% decrease, p ≤ 0.001), 1 μM (25% decrease, p ≤ 0.0001), 10 μM (38% decrease, p ≤ 0.0001) and 100 μM (22% decrease, p ≤ 0.0001) cocaine (Fig. 4b), Rab27A expression was significantly downregulated in exosomes after exposure to 100 nM (21% decrease, p ≤ 0.05), 1 µM (24% decrease, p ≤ 0.001) and 100 µM (23% decrease, p ≤ 0.001) cocaine (Fig. 4c), and Rab35 expression had a slight increase at 10 nM and 100 nM and then a declining trend which was not statistically significant (Fig. 4d). This data suggests that Rab proteins may have a role in the reduction of exosome particles per mL.

### Effects of Cocaine on the Lipid Components of Exosomes

**Fig. 5:**
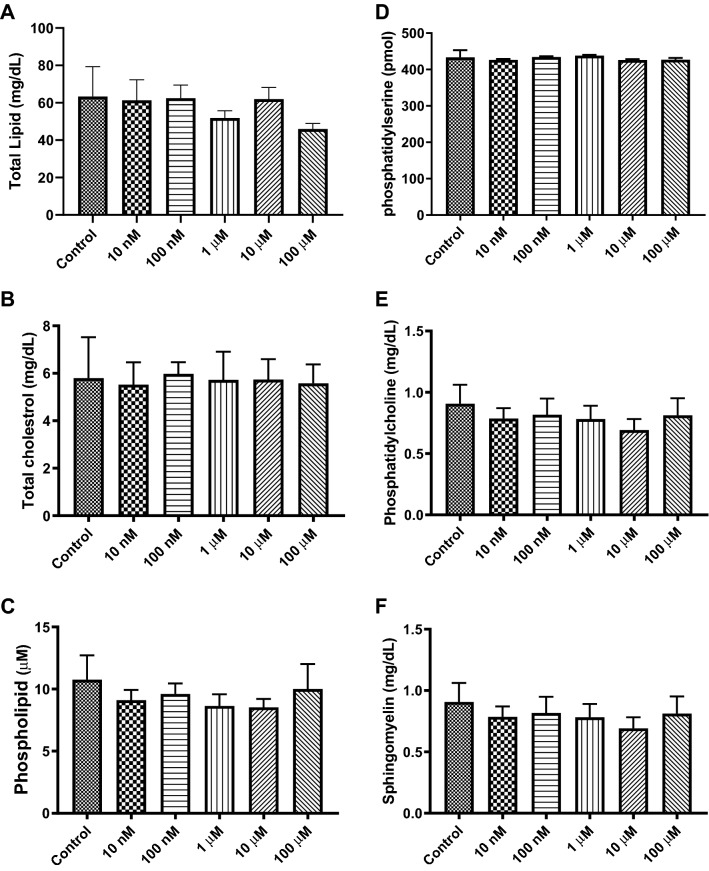
Effects of cocaine on exosomal lipids. To examine the expression of various important lipids in exosomes, cells were exposed to various concentrations of cocaine for 24 h, and lipid components were tested in exosomes. **a** total lipids, **b** total cholesterol, **c** phospholipids, **d** phosphatidylserine, **e** phosphatidylcholine and **f** sphingomyelin were determined in exosomes by ELISA-based fluorometric assays. Graph showed the mean of SD derived from 5 independent experiment in triplicates

Lipids are a diverse group of molecules that consist of monoglycerides, diglycerides, triglycerides, fats, sterols, and others. Lipids not only play important roles in the maintenance of cellular homeostasis and membrane integrity but also play significant roles in cellular communications, signaling, and apoptosis. To examine the distribution of lipids in exosomes, we performed fluorometric assays. Our data indicated that the expression levels of total lipids, phosphatidylcholine, phosphatidylserine, phospholipids, phosphatidylserine, sphingomyelin, and cholesterol were not significantly altered by exposure to cocaine (Fig. 5a–f).

## Discussion

Cocaine has been found to be associated with a variety of CNS disorders, such as the increased risk of stroke, seizures, cognitive impairments, depression, and, in extreme cases, death [48–50]. One plausible explanation for the many effects that cocaine has on the CNS may be the interruption of cell-to-cell communications and cell signaling, including those that involve microglial cells. Microglial cells are the most potent resident immune cells of the CNS and play a critical role in the surveillance of the brain environment [49, 51]. Although other studies have demonstrated that cocaine plays a role during endoplasmic reticulum stress, neuroinflammation, and Toll-like receptor-signaling, whether cocaine affects exosome biogenesis and composition has not been addressed until the current study. In previous studies, we examined the effects of another drug of abuse, alcohol, on exosome composition, and characteristics. Alcohol was found to have differential effects on the expression of several proteins in microglial cells [35], suggesting that other drugs of abuse could also affect microglial protein expression.

In the present investigation, we evaluated the cocaine-specific effects on the biogenesis and composition of BV2 microglial cell-derived exosomes. Exosomes are nanosized vesicles that originate from the fusion of MVBs with the plasma membrane and are composed of proteins, lipids, mRNAs, and miRNAs. Exosomes play crucial roles in cellular communications, signaling, and the transportation of various molecules [52–55]. Recent research has addressed the roles played by exosomes in CNS-associated disorders (neurodegenerative, neurodevelopmental, and neuroinflammatory disorders) and immune regulation, and their roles as therapeutic vesicles [56–61]; however, whether cocaine-mediated alterations occur in exosomes (in the contexts of biogenesis and composition) is not yet understood. A study by Carone et. al, evaluated the effect of cocaine on tunneling nanotube formation and extracellular vesicle release in glioblastoma cell cultures [62]. This study used a range of cocaine concentrations to evaluate the effects of cocaine on tunneling nanotube formation and exosomes produced from glioblastoma cells. Our study herein, used a similar range of concentrations and time points that overlap this study. Our findings suggested that exposure for 24 h to 100 µM cocaine significantly reduced the cell viability of BV2 microglial cells when compared with the control (Fig. 1a and b). Our findings also revealed that the mean size of exosomes after cocaine exposure remained unchanged (Fig. 1c and d), whereas the production of exosomes (particles per mL) was markedly reduced after exposure to 100 nM–10 µM cocaine compared with the control (Fig. 1d).

Cell membrane proteins, such as CD63 and CD81, which are tetraspanin molecules, interact with a variety of cell surface markers and intracellular molecules and are involved in adhesion, motility, membrane organization, and signal transduction [35, 63]. Moreover, CD11b (a surface marker for microglia, monocytes, and macrophages) and CD18 are transmembrane proteins that play critical roles in cellular adhesion [64]. In this study, we showed that the expression of CD11b and CD18 were significantly upregulated in BV2 cells after exposure to 100 µM cocaine (data not shown). These findings are in agreement with previous research that showed the increased expression of CD11b following nitric oxide exposure was associated with the activation of microglial cells during neurodegenerative inflammation [65]. A recent report has shown that disease-associated microglia express high levels of CD63, CD9, itgax, and Axl [66]. However, we found that CD63 did not demonstrate significant changes following cocaine exposure (Fig. 2). A significant downregulation was observed for CD81 expression after exposure to 100 nm, 1 µM, 10 µM, and 100 µM cocaine when compared with the control (data not shown). Furthermore, CD11b (Fig. 2b), CD18 (Fig. 2c), and CD63 (Fig. 2d) showed a slightly decreasing pattern of expression in exosomes, but these changes were not significant. CD81 was less expressed in microglial-derived exosomes (data not shown). These findings agreed with the previous studies and suggested that cocaine can impact the composition of exosomes.

Hsps are an evolutionarily conserved group of molecular chaperone proteins found in eukaryotes and prokaryotes and demonstrate protective functions under stress and trauma conditions, based on the upregulation of their expression levels under these conditions [67, 68]. Levandowski et al, in 2016, showed that cocaine addiction exerted stress during early life and accelerated the cellular aging process among women [69]. Our findings demonstrated that the expression levels of Hsp70 (Fig. 3a) and Hsp90 (Fig. 3b) have upregulated in BV2 cell-derived exosomes, suggesting that cocaine exerts stress on BV2 cells, which can further modulate exosome biogenesis and composition.

Rab proteins are well-known members of the Ras superfamily of small Rab GTPases, which play important roles during exosome biogenesis and secretion [46, 70]. Rab5 and Rab7 can be found in the plasma membrane and early endosomes and are associated with controlled trafficking, whereas Rab11, Rab27A, and Rab35 contribute to the sorting, secretion, and transportation of exosomes. Previous reports have indicated that Rab5 regulates the early endocytic pathway, can be found on clathrin-coated vesicles, and regulates endosomal trafficking [45, 47]. Rab7 is an important regulatory component of the endosome-to-lysosome pathway [47]; however, the present findings suggested that Rab7 expression was upregulated in exosomes after cocaine exposure (Fig. 4a), suggesting that it may be involved in directing exosomes toward the lysosomal degradation pathway, resulting in a decrease in the number of particles per mL. Each Rab protein has a specific subcellular localization and a different function; however, only Rab11, Rab27, and Rab35 are known to regulate exosome release/secretion [71]. We showed that Rab11 (Fig. 4b) and Rab27 (Fig. 4c) expression in exosomes were significantly suppressed compared with their respective controls (Fig. 4). These findings suggested that the downregulation of Rab protein expression might be associated with reduced exosome release into the extracellular environment; therefore, the observed decrease in exosome particles per mL can be correlated with the current findings (Fig. 1e). Since Rab proteins are implemented in multiple aspects of disease progression, they might represent new therapeutic targets in controlling disease progression [72]. Although Rab-specific drugs have not been available for public use, it is important to add findings that exposure to cocaine may regulate Rab proteins. Rab-specific modulation has already been reviewed by Qin et al. demonstrated the use of nucleotide based competitive inhibitors that target kinases, blocking protein–protein interactions, and small interfering RNA such as siRNA and miRNA [73].

Lipids are the most important components of the plasma membrane and play important roles in cellular homeostasis, membrane integrity, cellular communications, signaling, and apoptosis. Studies have demonstrated that exosomes are enriched in lipids compared with their parent cells, and we hypothesized that drugs of abuse, such as cocaine, may affect the lipid composition of exosomes [74–76]; however, our results indicated that the expression levels of total lipids, phosphatidylcholine, phosphatidylserine, phospholipids, phosphatidylserine, sphingomyelin, and cholesterol remained unchanged during exosome production and secretion after cocaine exposure (Fig. 5). One caveat to our findings may be the limited range of sensitivity of our detection system. Further lipid analysis might be warranted with an assay that has a greater sensitivity (i.e. gas chromatography-mass spectrometry).

The strength of this study is that it adds to the body of literature concerning the effect of cocaine on exosome production in microglial cells. To our knowledge this is the first study of its kind to evaluate measures such as microglia cell viability after cocaine treatment, exosome size, exosome quantity, and composition (i.e. protein and lipid quantity/ profile). Although, one limitation of our work is that our work cannot be compared directly to Carone et al. [62], because the experimental design differs. However, some of our findings concerning the effects of cocaine on total exosomes numbers after time show opposite results on Carone’s study, indicated that the effect of cocaine and exosome regulation may be cell-type specific. In our study, we focused on the effect of cocaine on proteins and lipids directly and did not look at the effect of microRNA. Cocaine could plausibly effect microRNA content and will be the focus of future work.

## Conclusions

In summary, our findings provide insight into cocaine-specific effects on BV2 cell-derived exosome biogenesis and composition. In brief, our findings demonstrated that high concentrations of cocaine exposure reduced the cell survival of microglial BV2 cells and disturbed exosome biogenesis and composition by modulating the expression levels of Rab GTPases and membrane proteins, such as CD molecules, Hsps, and signaling molecules; however, the lipid components in exosomes remained unchanged after cocaine exposure. Specifically, increasing Rab7 expression could lead to increase clearance of exosomes via lysosomes and a concomitant decrease in exosome production by regulating Rab11 and Rab27. Therefore, our findings suggested that cocaine can have dramatic effects on exosome biogenesis and composition; however, further investigation is warranted to explore the specific underlying mechanism(s).

## Supplementary Information

Below is the link to the electronic supplementary material.

Electronic supplementary material 1 (PPTX 20984 kb)

Electronic supplementary material 2 (DOCX 16 kb)
