# Supplementary material for: Cocaine‐Specific Effects on Exosome Biogenesis in Microglial Cells
Source: Neurochem Res. 2021 Feb 8;46(4):1006–18. doi: 10.1007/s11064-021-03231-2 (PMC7946671; doi:10.1007/s11064-021-03231-2)
Supplement: Supplementary file 1 — Electronic supplementary material 1 (PPTX 20984 kb) [file 11064_2021_3231_MOESM1_ESM.pptx]

## Slide 1
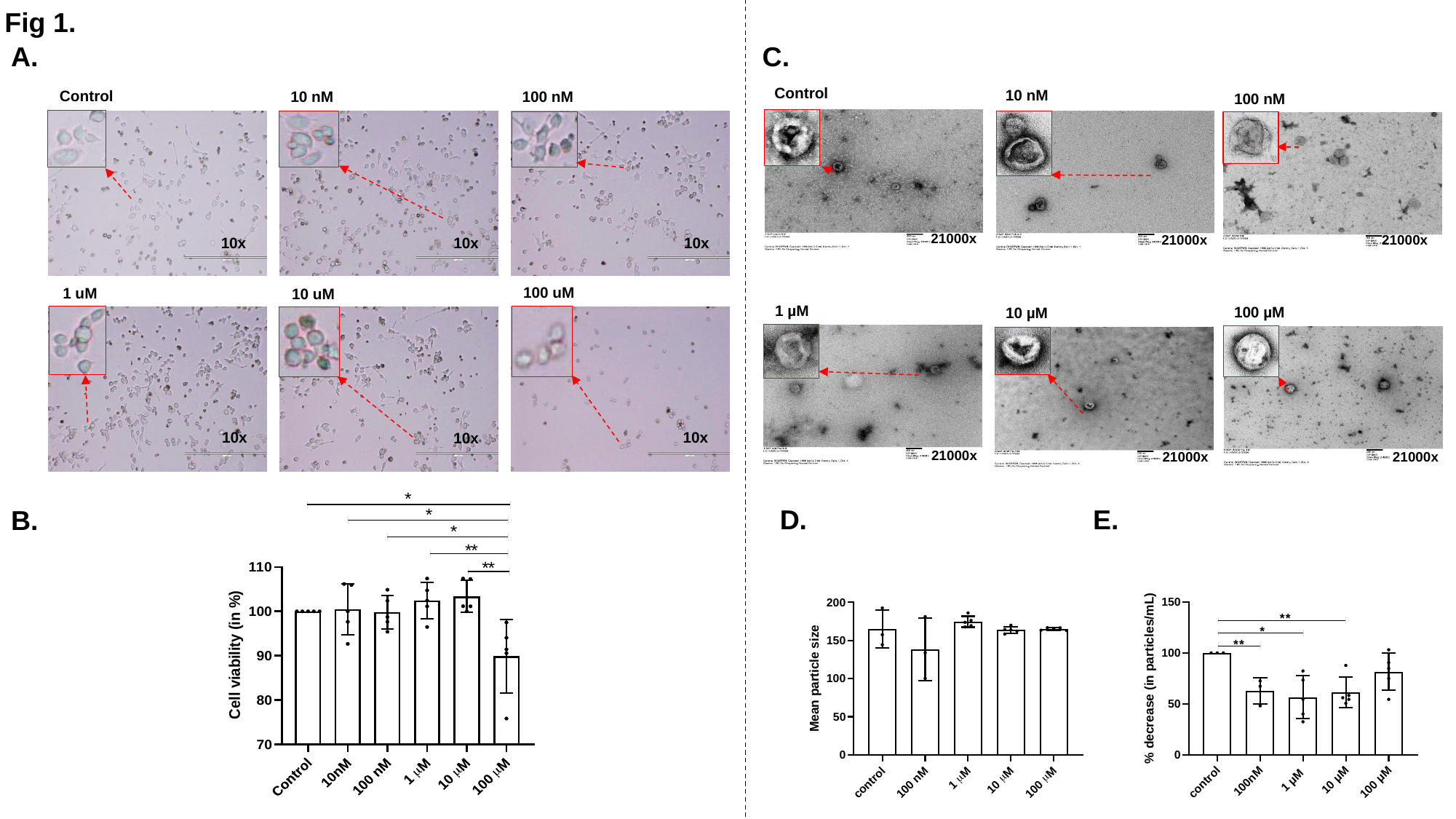

Fig 1.
A.
C.
Control
10 nM
Control
10 nM
100 nM
100 nM
21000x
21000x
21000x
10x
10x
10x
100 uM
1 uM
10 uM
1 µM
100 µM
10 µM
10x
10x
10x
21000x
21000x
21000x
D.
E.
B.

## Slide 2
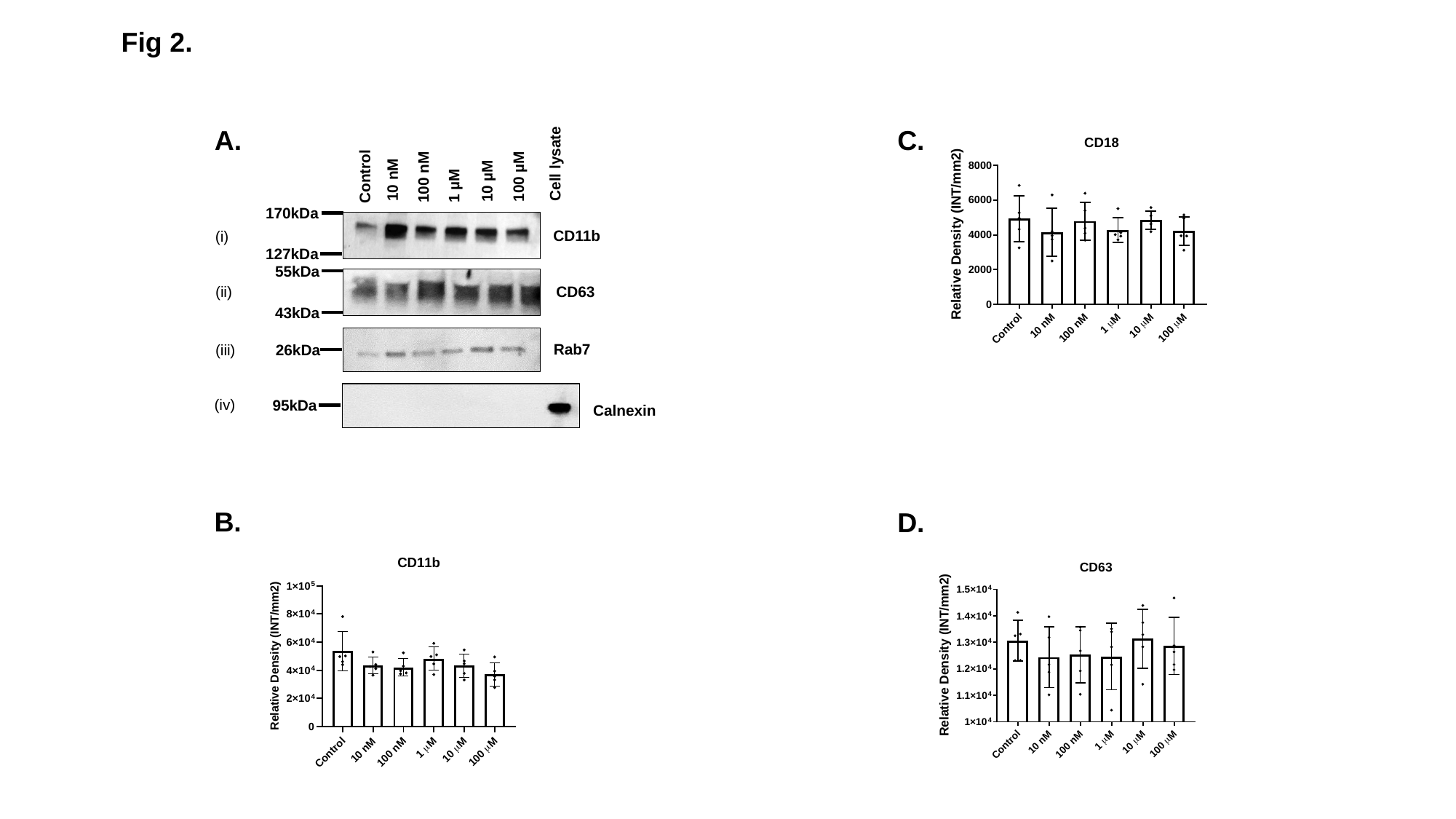

Fig 2.
C.
A.
100 µM
Cell lysate
10 µM
100 nM
Control
10 nM
1 µM
170kDa
CD11b
(i)
127kDa
55kDa
(ii)
CD63
43kDa
Rab7
26kDa
(iii)
(iv)
95kDa
Calnexin
B.
D.

## Slide 3
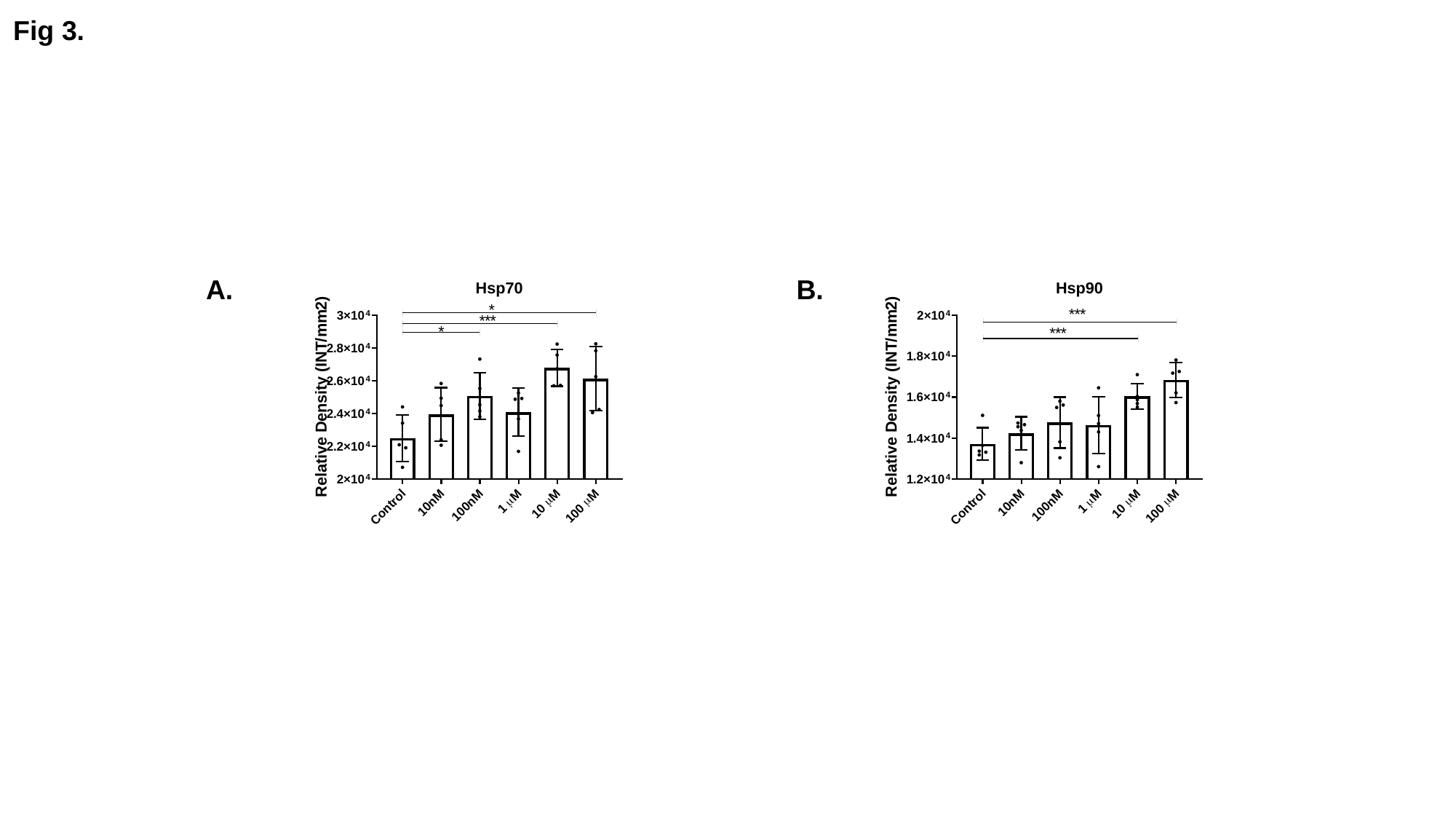

Fig 3.
A.
B.

## Slide 4
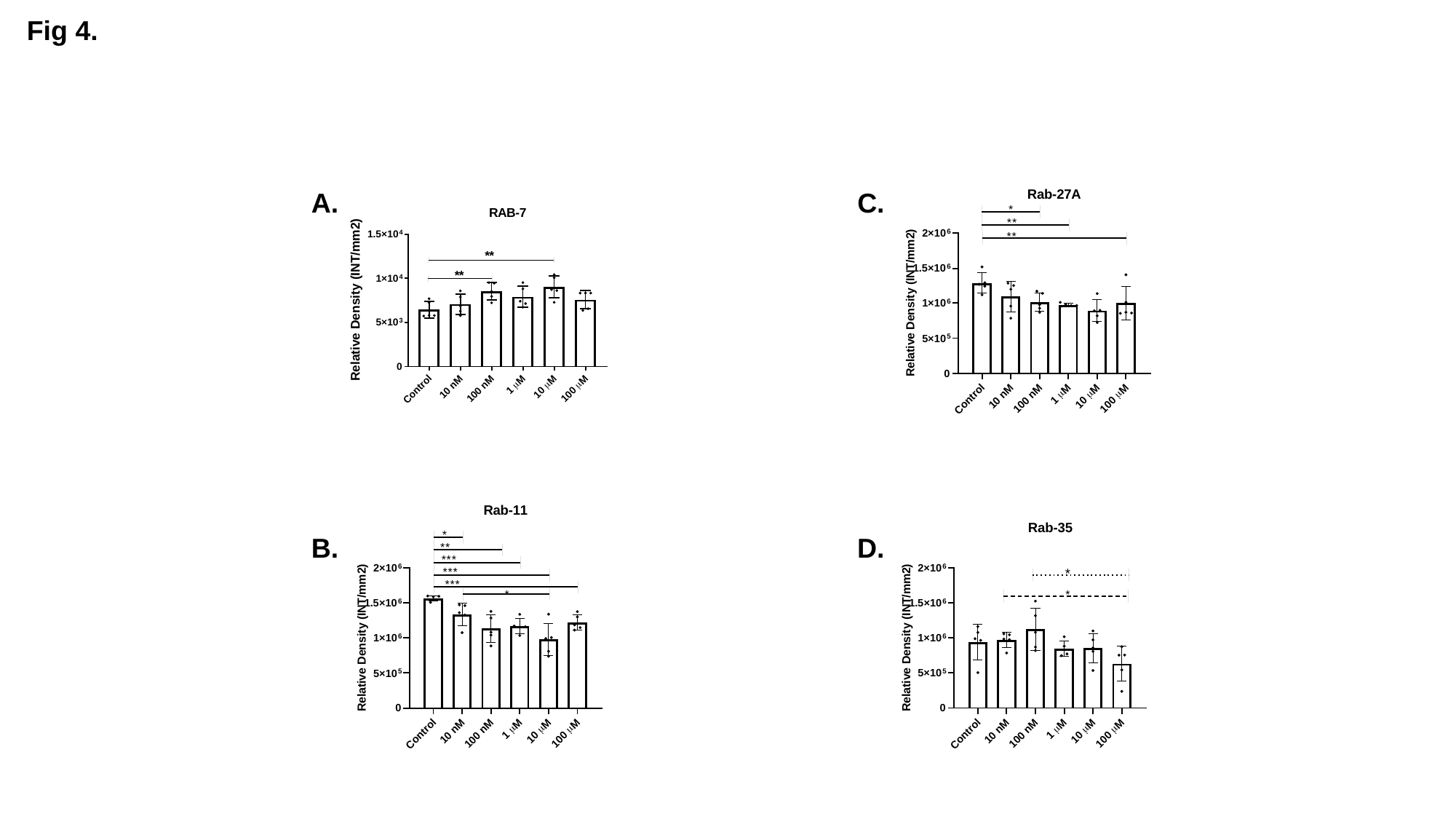

Fig 4.
C.
A.
B.
D.

## Slide 5
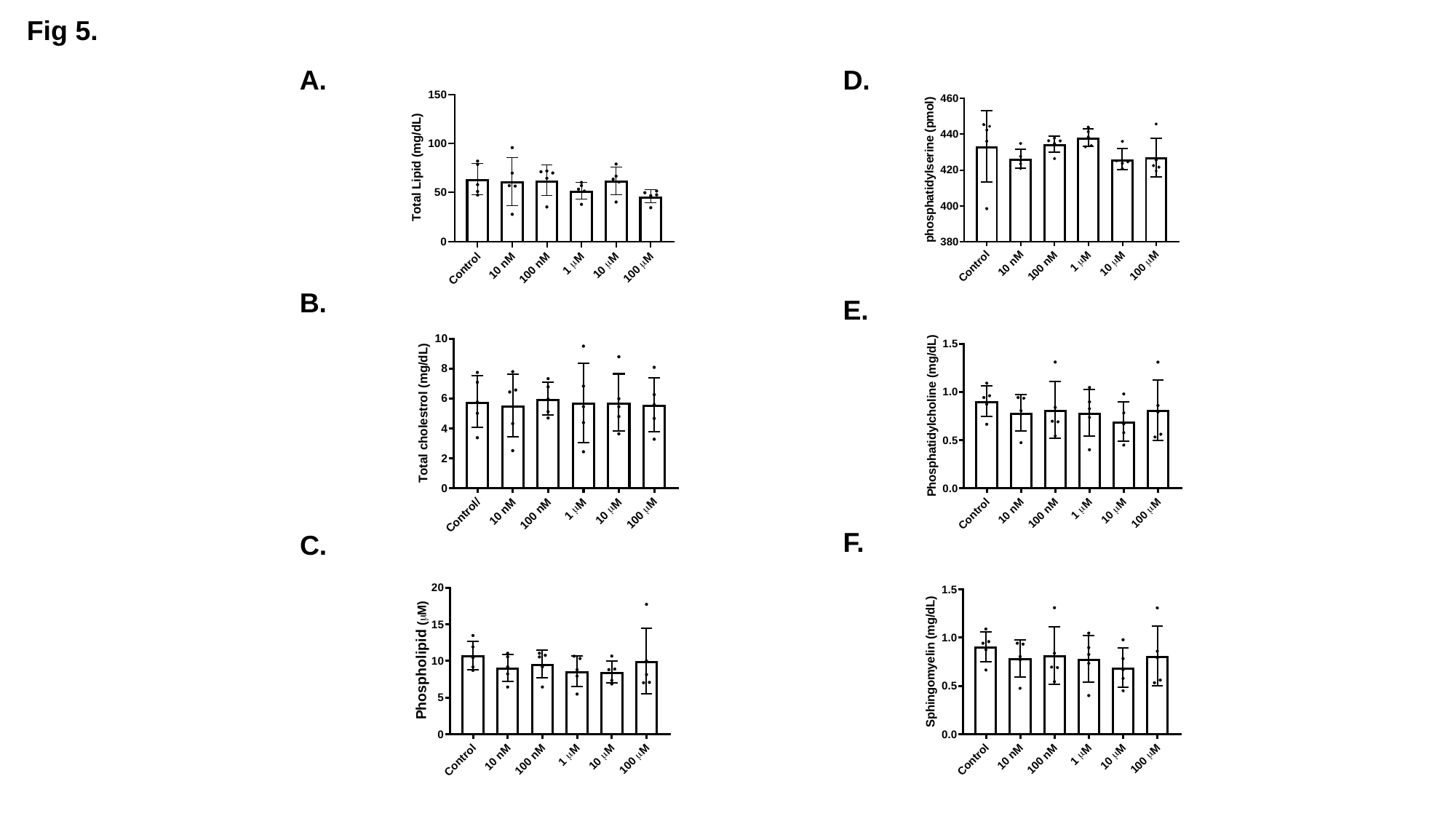

Fig 5.
A.
D.
B.
E.
F.
C.
