# Supplementary material for: Cocaine‐Specific Effects on Exosome Biogenesis in Microglial Cells
Source: Neurochem Res. 2021 Feb 8;46(4):1006–18. doi: 10.1007/s11064-021-03231-2 (PMC7946671; doi:10.1007/s11064-021-03231-2)
Supplement: Supplementary file 2 — Electronic supplementary material 2 (DOCX 16 kb) [file 11064_2021_3231_MOESM2_ESM.docx]

**Supplemental Figure Legends**

**Supl. fig. 1** Cocaine-specific effects on BV2 microglial cell viability and the mean size and number of particles. BV2 microglial cells were treated with 10 nM, 100 nM, 1 µM, 10 µM, and 100 µM cocaine. Cells were grown in exosome-free medium and the cocaine was added for a maximum of 24 h. A.) Microscopy, B.) Cell Viability, C.) TEM, D.) Mean particle size and E.) Particle/mL. Mean size is shown in nanometers, and particle numbers are shown as 10^8^ per mL. Statistical significance is taken from 3-5 independent experiment in triplicates and indicated the mean of SD as follows: *, p < 0.05; **, p <0.001; and ***, p < 0.0001 (individual data points have been shown for supplemental figure 1).

**Supl. fig. 2** Cocaine-specific effects on cell membrane molecules. BV2 microglial cells were treated with cocaine (10 nM, 100 nM, 1 µM, 10 µM, and 100 µM) for 24 h, and the expression of cell membrane molecules in exosomes was determined using western and dot blot analysis. A.) representative western blots; i.) CD11b, ii.) CD63, iii.) Rab7 and iv.) Calnexin, B.) CD11b expression, C.) CD18 expression and D.) CD63 expression. Statistical significance is taken from 5 independent experiment in triplicates and indicated the mean of SD as follows: *, p < 0.05; and **, p < 0.001 (individual data points have been shown for supplemental figure 2).

**Supl. fig. 3** Cocaine-specific effects on HSPs in BV2 cells and exosomes. BV2 microglial cells were treated with cocaine (10 nM, 100 nM, 1 µM, 10 µM, and 100 µM) for 24 h, and the expression levels of was evaluatedin in exosomes. A.) Hsp70 and B.) Hsp90 densities derived from dot blot. Statistical significance is derived from 5 independent experiment in triplicates is indicated the mean of SD as follows: *, p < 0.05; **, p < 0.001; and ***, p < 0.0001 (individual data points have been shown for supplemental figure 3).

**Supl. fig. 4** Cocaine-specific effects on Rab GTPases. To examine the expression of Rab GTPases in microglial cells, cells were incubated with 10 nM, 100 nM, 1 µM, 10 µM, and 100 µM cocaine for 24 h, and Rab protein expression levels were evaluated in BV2 cells and exosomes using dot blot analysis. A.) Rab7, B.) Rab11, C.) Rab27A and D.) Rab35 expression in BV2 cell-derived exosomes. Statistical significance derived from 5 independent experiment in triplicates is indicated the mean of SD as follows: *, p < 0.05; **, p < 0.001; and ***, p < 0.0001 (individual data points have been shown for supplemental figure 4).

**Supl. fig. 5** Effects of cocaine on exosomal lipids. To examine the expression of various important lipids in exosomes, cells were exposed to various concentrations of cocaine for 24 h, and lipid components were tested in exosomes. A.) total lipids, B.) total cholesterol, C.) phospholipids, D.) phosphatidylserine, E.) phosphatidylcholine and F.) sphingomyelin were determined in exosomes by ELISA-based fluorometric assays. Graph showed the mean of SD derived from 5 independent experiment in triplicates (individual data points have been shown for supplemental figure 5).
